# Fumarate and fumarate hydratase: an immunometabolite regulator of inflammation and diseases

**DOI:** 10.3389/fimmu.2026.1756976

**Published:** 2026-07-15

**Authors:** Zewen Jiang, Yujian Zhong, Zhuokun He, Ziqi He, Wei Wang, Ruyuan He

**Affiliations:** 1 Department of Orthopedic Surgery, Renmin Hospital of Wuhan University, Wuhan University, Wuhan, Hubei, China; 2 Department of Cardiovascular Surgery, Renmin Hospital of Wuhan University, Wuhan University, Wuhan, Hubei, China; 3 Department of Urology, Renmin Hospital of Wuhan University, Wuhan University, Wuhan, Hubei, China; 4 Department of Thoracic Surgery, Renmin Hospital of Wuhan University, Wuhan University, Wuhan, Hubei, China

**Keywords:** chronic inflammatory diseases, epigenetics, fumarate, fumarate hydratase (FH), immunometabolism, inflammation, succination

## Abstract

Once regarded solely as an intermediate of the tricarboxylic acid (TCA) cycle involved in energy production, fumarate has now emerged as a pivotal immunometabolite with far-reaching effects on inflammatory signaling and immune cell fate. This review comprehensively delineates the dual nature of fumarate, which functions as a context-dependent rheostat of inflammation. Intracellular fumarate levels are tightly regulated by enzymatic activity, transport systems, and exogenous sources, including the pharmacological agent dimethyl fumarate (DMF). Fumarate can covalently modify critical cysteine residues in proteins through a process known as succination. Importantly, DMF acts at supraphysiological concentrations and may engage mechanisms distinct from those associated with endogenously accumulated fumarate. This unique post-translational modification enables fumarate to directly modulate key signaling pathways, including nuclear factor kappa B (NF-κB), nuclear factor erythroid 2-related factor 2 (NRF2), hypoxia-inducible factor 1-alpha (HIF-1α), Janus kinase/signal transducer and activator of transcription (JAK-STAT), and the NLR family pyrin domain-containing 3 (NLRP3) inflammasome, thereby orchestrating a broad anti-inflammatory program. We further examine how fumarate reshapes the functional phenotypes of macrophages, dendritic cells, T cells, and B cells, ultimately skewing immune responses toward tolerance and resolution. Crucially, this review distinguishes among the physiological roles of endogenous fumarate, the pathological consequences of fumarate accumulation resulting from fumarate hydratase (FH) deficiency, and the pharmacological actions of exogenous fumarate esters. Conversely, dysregulated fumarate metabolism, as observed in conditions such as hereditary leiomyomatosis and renal cell carcinoma (HLRCC) and systemic lupus erythematosus, can paradoxically promote pathological inflammation. The successful clinical translation of fumarate esters, particularly DMF, for the treatment of multiple sclerosis and psoriasis underscores their therapeutic potential. By synthesizing recent advances in fumarate biology, this review not only elucidates its role as a fundamental link between cellular metabolism and immunity but also highlights future directions for targeting fumarate-associated pathways in a broad spectrum of chronic inflammatory diseases.

## 1 Introduction

Fumarate, a key intermediate of the TCA cycle, has long been recognized for its essential role in cellular energy metabolism. However, accumulating evidence has expanded its functional repertoire beyond bioenergetics, positioning fumarate as a critical immunometabolic mediator involved in the regulation of inflammatory signaling and immune cell function. This review comprehensively examines the multifaceted roles of fumarate, ranging from its biochemical synthesis and metabolic regulation to its profound effects on inflammatory pathways and the pathogenesis of chronic diseases.

Intracellular fumarate levels are tightly regulated through multiple mechanisms, including enzymatic activity within the TCA and urea cycles, transport systems, and exogenous sources such as the widely used therapeutic agent DMF. Notably, fumarate can covalently modify proteins through a process termed succination, resulting in functional alterations of key signaling molecules and transcription factors. These modifications exert broad anti-inflammatory effects by influencing inflammasome activation, cytokine production, and immune cell differentiation. Furthermore, fumarate modulates the behavior of macrophages, dendritic cells, and T lymphocytes, thereby shaping both innate and adaptive immune responses.

Dysregulation of fumarate metabolism has been implicated in several chronic inflammatory and autoimmune disorders. The therapeutic potential of fumarate-based compounds, particularly DMF, has been validated in clinical settings, underscoring their efficacy in modulating immune activity. However, the pharmacological actions of DMF, an electrophilic drug that rapidly depletes glutathione, are not directly equivalent to the effects resulting from endogenous fumarate accumulation. This review synthesizes current knowledge regarding the metabolic, signaling, and immunoregulatory functions of fumarate, emphasizing its dual role as both a driver and suppressor of inflammation in a context-dependent manner. In addition, this review addresses the critical distinction among the physiological functions of endogenous fumarate, the pathological consequences arising from FH deficiency-driven fumarate accumulation, and the pharmacological effects of exogenous fumarate esters, including DMF and monomethyl fumarate (MMF), which exhibit distinct metabolic and functional properties. It also discusses current clinical applications and future research directions aimed at harnessing fumarate-mediated pathways for the treatment of immune-mediated diseases.

## 2 Metabolic role of fumarate

### 2.1 Biochemical structure and function of fumarate in the TCA cycle

Fumarate is a four-carbon dicarboxylic acid with the chemical formula C_4_H_4_O_4_. Its molecular structure contains a double bond between two carbon atoms, each of which is bonded to a carboxyl group (COOH). The systematic name of fumarate is trans-butenedioic acid, reflecting the trans configuration of the double bond between the two carboxyl groups (1).

Fumarate plays a pivotal role in the tricarboxylic acid (TCA) cycle, also known as the Krebs cycle, which serves as a central metabolic pathway responsible for energy production through the oxidation of acetyl-CoA derived from carbohydrates, lipids, and proteins (2). Fumarate is generated from succinate through the catalytic activity of succinate dehydrogenase (SDH), which also functions as complex II of the electron transport chain. During this oxidation reaction, flavin adenine dinucleotide (FAD) is reduced to flavin adenine dinucleotide reduced form (FADH^2^) (3). Subsequently, fumarate is hydrated to malate by fumarate hydratase (FH). This reaction is essential for continuation of the TCA cycle and the eventual regeneration of oxaloacetate. The primary function of the TCA cycle is to generate high-energy electron carriers, including nicotinamide adenine dinucleotide (NADH) and FADH^2^, which subsequently fuel the electron transport chain to produce adenosine triphosphate (ATP), the principal energy currency of the cell (4). Through participation in these metabolic reactions, fumarate contributes substantially to the maintenance of cellular energy homeostasis.

### 2.2 Dual subcellular localization of FH

FH is localized in both the mitochondria and cytosol of human cells, a phenomenon evolutionarily conserved from yeast to humans. In humans, this dual localization is achieved through alternative transcription initiation from a single gene (5). A broad promoter generates multiple messenger RNA (mRNA) transcripts with distinct 5’ untranslated regions. Transcripts containing a sufficiently long 5’ leader sequence encode a longer precursor protein harboring a mitochondrial targeting sequence (MTS), thereby enabling mitochondrial import. In contrast, transcripts initiated closer to the start codon lack a functional MTS and are translated from a downstream start codon, resulting in a shorter isoform that remains in the cytosol (5). Cytosolic FH has been implicated in DNA damage repair and epigenetic regulation.

### 2.3 Fumarate as a metabolic intermediate beyond energy production

Beyond its established role in energy metabolism, fumarate has increasingly been recognized as a signaling metabolite. Accumulation of fumarate, whether resulting from metabolic rewiring or FH deficiency, facilitates non-enzymatic post-translational modifications through succination and competitively inhibits α-ketoglutarate (α-KG)-dependent dioxygenases. These mechanisms functionally connect TCA cycle activity with epigenetic regulation and inflammatory signaling pathways.

## 3 Regulation of intracellular fumarate levels

Intracellular fumarate concentrations are tightly regulated by multiple metabolic and enzymatic processes, as well as by extracellular factors and cellular physiological conditions. The principal pathways and mechanisms involved in this regulation are summarized below.

### 3.1 Tricarboxylic acid cycle

**Figure 1:**
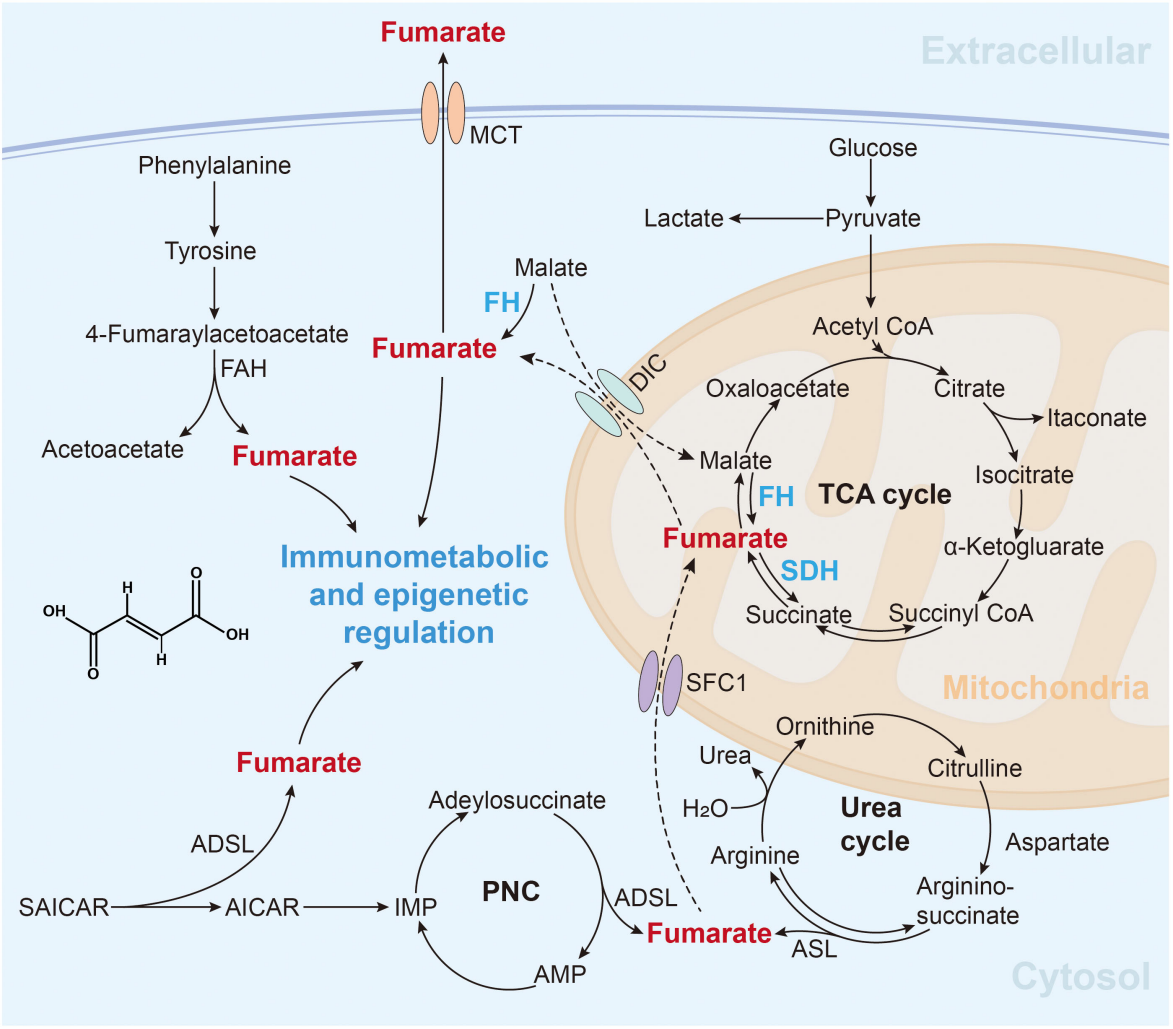
Fumarate as a central metabolic hub integrating the TCA cycle, urea cycle, purine nucleotide synthesis, and immunometabolic regulation. This schematic depicts the multifunctional role of fumarate, a key intermediate that links disparate metabolic pathways. Fumarate is generated from multiple sources: (1) within the TCA cycle, via succinate dehydrogenase (SDH)-mediated oxidation of succinate and via fumarate hydratase (FH)-catalyzed dehydration of malate; (2) from tyrosine/phenylalanine catabolism through 4-fumarylacetoacetate cleavage by fumarylacetoacetate hydrolase (FAH); (3) from the urea cycle via argininosuccinate lyase (ASL), which produces fumarate together with arginine; and (4) from purine metabolism via adenylosuccinate lyase (ADSL), which cleaves adenylosuccinate to AMP and fumarate. Conversely, FH also converts fumarate to malate, replenishing the TCA cycle. Fumarate can be exported across membranes, possibly via monocarboxylate transporters (MCT), and contributes to immunometabolic and epigenetic regulation-often by inhibiting α-ketoglutarate-dependent dioxygenases, thereby influencing gene expression and cellular metabolism. The diagram also illustrates interactions with other metabolites, including itaconate, acetoacetate, citrate, isocitrate, α-ketoglutarate, succinate, succinyl-CoA, as well as intermediates of the urea cycle (ornithine, citrulline) and purine synthesis (SAICAR, AICAR, IMP). Key enzymes and complexes are labeled (FH, SDH, FAH, ASL, ADSL, SFC1).

Fumarate is synthesized as an intermediate metabolite within the TCA cycle. Its formation from succinate is catalyzed by SDH, a critical enzymatic component that additionally participates in the electron transport chain. SDH activity is modulated by numerous cellular and molecular factors, including the intracellular redox state, epigenetic alterations, transcriptional regulation, and post-translational modifications (6–8). Impairment of SDH activity, whether caused by genetic mutations or pharmacological inhibition, may lead to succinate accumulation and reduced fumarate production. Similarly, the catalytic activity of FH is subject to regulatory mechanisms that influence the conversion rate of fumarate to malate (**Figure 1**). Consequently, diminished FH activity can result in intracellular fumarate accumulation (9).

### 3.2 Urea cycle

Fumarate is also generated during the urea cycle, primarily in hepatocytes, through cleavage of argininosuccinate catalyzed by argininosuccinate lyase (ASL). This reaction contributes significantly to the intracellular fumarate pool, particularly in tissues characterized by active amino acid metabolism (10). Cytosolic fumarate may subsequently be transported into mitochondria through specific membrane transporters, particularly the succinate/fumarate carrier (SFC1) (11, 12). Such transport mechanisms facilitate metabolic integration between ureagenesis and the TCA cycle (**Figure 1**). In addition, fumarate is produced by fumarylacetoacetate hydrolase (FAH), a key enzyme involved in tyrosine and phenylalanine catabolism. Located in the cytosol, FAH catalyzes the hydrolytic cleavage of fumarylacetoacetate, breaking a carbon-carbon bond to generate acetoacetate and fumarate (13).

### 3.3 Exogenous sources and factors

Dietary intake and supplementation involving fumarate or its metabolic precursors may influence intracellular fumarate concentrations. Furthermore, fumarate is generated through several ancillary biosynthetic pathways. One notable example is *de novo* purine biosynthesis, in which adenylosuccinate lyase (ADSL) catalyzes fumarate release during cleavage of succinylaminoimidazolecarboxamide ribotide (SAICAR) (14). The same enzyme also catalyzes the conversion of adenylosuccinate into either 5-aminoimidazole-4-carboxamide ribotide or adenosine monophosphate (AMP). These reactions proceed through a concerted general acid-general base catalytic mechanism that facilitates β-elimination of fumarate, thereby contributing to elevated intracellular fumarate levels. Given the indispensable role of ADSL in DNA replication, fumarate metabolism may represent an important biochemical link between nucleotide biosynthesis and broader metabolic networks (15). Additionally, certain pharmacological compounds, including DMF, which is widely used in the treatment of multiple sclerosis (MS), can be metabolized to increase intracellular fumarate levels (16, 17).

### 3.4 Intracellular and extracellular transport mechanisms

Fumarate is generated within mitochondria as part of the TCA cycle. However, its distribution across intracellular compartments and between cells is regulated through multiple transport mechanisms that maintain metabolic homeostasis. Initially, fumarate is transported from the mitochondrial matrix into the cytosol primarily by dicarboxylate carriers (DICs) located in the inner mitochondrial membrane. These transport proteins mediate antiport exchange between fumarate and other dicarboxylates, including succinate and malate (18). Another important mechanism facilitating fumarate trafficking is the malate-aspartate shuttle. Within this pathway, mitochondrial fumarase catalyzes the conversion of fumarate to malate, which is subsequently exported into the cytosol. Cytosolic fumarase can then regenerate fumarate from malate, thereby coupling mitochondrial and cytoplasmic metabolic fluxes (19). Under specific conditions, such as oxidative stress or metabolic dysregulation, fumarate may also be exported extracellularly. This process is facilitated by monocarboxylate transporters (MCTs), which mediate the exchange of fumarate with extracellular anions, including lactate (**Figure 1**). Such transport mechanisms may help alleviate intracellular acidification during metabolic stress (20). The precise subcellular localization of fumarate production and its downstream molecular targets is critical for understanding its context-dependent immunometabolic functions, particularly in light of the distinct mitochondrial and cytosolic FH isoforms.

### 3.5 Distinguishing three contexts: endogenous fumarate, FH deficiency, and pharmacological fumarates

It is essential to distinguish among three distinct contexts in which fumarate modulates immune responses. The first involves endogenous fumarate accumulation resulting from metabolic rewiring, such as that observed in lipopolysaccharide (LPS)-activated macrophages. In this setting, fumarate accumulation is typically transient, compartmentalized, and maintained at moderate concentrations. The second context involves genetic FH deficiency, such as that observed in hereditary leiomyomatosis and renal cell carcinoma (HLRCC), which leads to chronic and markedly elevated fumarate levels associated with extensive metabolic and epigenetic remodeling. The third context involves pharmacological fumarate esters, including DMF and monomethyl fumarate (MMF), which are administered exogenously at supraphysiological concentrations, rapidly deplete intracellular glutathione (GSH), and modify numerous cysteine residues throughout the proteome. These three contexts differ fundamentally with respect to fumarate concentration, duration of exposure, subcellular localization, and downstream molecular targets. Throughout this review, we explicitly specify the biological context to which each finding pertains. Importantly, the pharmacological effects of exogenous fumarate esters, such as DMF and MMF, may involve mechanisms that differ from, or extend beyond, those associated with endogenous fumarate accumulation because of their strong electrophilic properties and capacity to rapidly deplete intracellular GSH. As emphasized by Cheng et al., esterified fumarate derivatives exhibit enhanced cellular permeability but may not fully recapitulate the biological effects of endogenous fumarate. Indeed, partial discrepancies between fumarate esters and naturally accumulated fumarate have been reported, highlighting the importance of validating findings through direct genetic or pharmacological manipulation of FH itself (21). Therefore, observations derived exclusively from DMF/MMF treatment should not be automatically extrapolated to the physiological or pathophysiological roles of endogenous fumarate unless supported by independent experimental approaches, such as FH inhibition or genetic FH deletion.

## 4 Fumarate in inflammation

### 4.1 Fumarate drive protein succination and inflammatory signaling pathways

**Figure 2:**
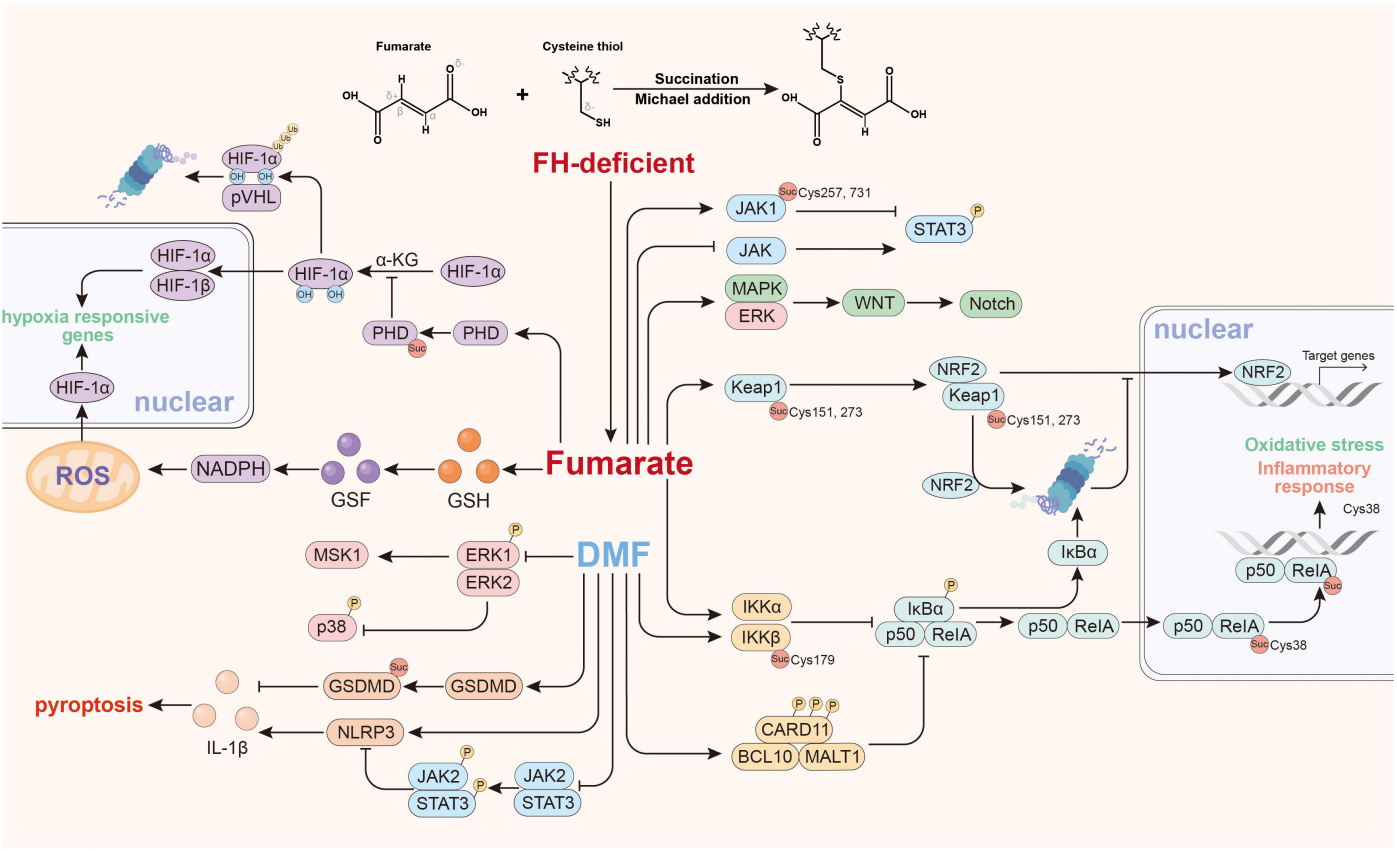
Fumarate and DMF as a signaling hub regulating hypoxia response, oxidative stress, inflammation, and pyroptosis. In fumarate hydratase (FH)-deficient contexts, fumarate accumulates and acts as an electrophilic metabolite that modifies cysteine residues in proteins via succination, a Michael addition reaction with cysteine thiols. Succination of key regulatory proteins, including PHD, Keap1, JAK1/2, STAT3, IKKβ, and GSDMD, alters multiple signaling pathways. FH deficiency stabilizes HIF-1α by inhibiting prolyl hydroxylase domain (PHD) enzymes, leading to upregulation of hypoxia-responsive genes in the nucleus. Fumarate also modulates redox homeostasis by depleting glutathione (GSH) and generating reactive oxygen species (ROS), activating NRF2-dependent antioxidant responses. Additionally, succination triggers inflammatory and pyroptotic pathways via NF-κB, NLRP3 inflammasome activation, and GSDMD cleavage, leading to IL-1β release. Parallel signaling through MAPK/ERK and WNT/Notch pathways integrates stress and proliferative signals. Therapeutic modulation with dimethyl fumarate (DMF) influences ERK1/2 and MSK1 phosphorylation, highlighting potential intervention points. Collectively, this schematic illustrates how fumarate accumulation and DMF orchestrate metabolic, oxidative, and inflammatory responses through cysteine succination, linking metabolic dysfunction to cellular stress and signaling networks.

Fumarate exhibits a unique characteristic among oncometabolites because of its chemical structure, which enables spontaneous reactions with thiol groups present in free cysteines or cysteine residues within glutathione (GSH) and proteins. This process, termed succination (**Figure 2**), generates post-translational modifications that alter the activity of multiple proteins implicated in the tumorigenesis of FH-deficient renal cell carcinoma (FHd-RCC) (22). his review specifically focuses on the effects of fumarate on several representative inflammatory signaling pathways through its capacity to form cysteine adducts and succinated protein modifications.

#### 4.1.1 NF-κB pathway

NF-κB is a central transcription factor governing inflammatory responses. Fumarate has been shown to modulate NF-κB signaling through both direct and indirect mechanisms. Experimental evidence demonstrates that both endogenous fumarate accumulation, such as that observed in FH-deficient cells, and exogenous DMF treatment induce succination of inhibitor of κB kinase β (IKKβ) at cysteine 179 within its activation loop. This modification impairs kinase activity and subsequently reduces phosphorylation and degradation of IκBα. As a result, NF-κB subunits, including p65 and p50, remain sequestered in the cytoplasm, thereby attenuating their transcriptional activity (23). Furthermore, ​​studies have shown that fumarate directly modifies the p65/RelA subunit at Cys38, a residue critical for DNA binding, disrupting its ability to recognize κB motifs in the promoters of pro-inflammatory genes. This leads to suppressed expression of cytokines including interleukin-6 (IL-6), tumor necrosis factor-alpha (TNF-α), and interleukin-1 beta (IL-1β) (24). In addition to directly targeting NF-κB signaling, fumarate also modulates upstream signaling complexes. For example, DMF inhibits the CARD11-BCL10-MALT1 (CBM) complex through succination, thereby attenuating NF-κB activation in human T-cell leukemia virus type 1 (HTLV-1)-infected T cells and limiting their proliferative and inflammatory capacities (25). Moreover, fumarate effectively activates the NRF2 pathway, which intersects with NF-κB signaling at multiple levels, including KEAP1 succination, redox modulation, and transcriptional cross-talk (26, 27).

#### 4.1.2 NRF2 pathway

As previously established, fumarate esters function as potent activators of the NRF2 antioxidant pathway through covalent modification of Kelch-like ECH-associated protein 1 (KEAP1) via succination (28, 29). Keap1, which acts as a cytoplasmic repressor of Nrf2, contains several redox-sensitive cysteine residues, including Cys151, C273, Cys288, and Cys319. Succination at residues such as Cys151 and Cys273 interferes with Keap1’s capacity to promote ubiquitination of Nrf2, facilitating the translocation of Nrf2 into the nucleus (30). Once activated, Nrf2 orchestrates the transcription of genes involved in mitigating oxidative stress and inflammation, such as heme oxygenase-1 (HO-1), enzymes responsible for GSH synthesis, and various detoxifying proteins. In immune cells, Nrf2 activation promotes a shift toward anti-inflammatory phenotypes; in macrophages, for instance, it suppresses secretion of IL-1β and IL-6 while enhancing production of interleukin-10 (IL-10) (31). DMF, the most extensively characterized fumarate derivative, has received FDA approval for clinical use. Its efficacy is closely tied to modulation of the Keap1-NRF2 axis, with studies identifying three distinct binding sites on Keap1 for DMF (32). MMF, the active metabolite of DMF, selectively modifies Cys151 on Keap1 and induces a concentration-dependent upregulation of Nrf2 in human and rodent astrocytes (33). Although the therapeutic benefits of DMF in MS and psoriasis are largely ascribed to Nrf2-mediated cytoprotection, it is noteworthy that certain anti-inflammatory effects persist even in the absence of Nrf2, indicating involvement of alternative mechanisms (34). It should be emphasized that these findings are derived from pharmacological DMF treatment (supraphysiological concentrations, electrophilic stress) and may not fully recapitulate the effects of endogenous fumarate accumulation, which occurs at lower concentrations and within specific subcellular compartments.

#### 4.1.3 HIF-1α pathway

HIF-1α, the oxygen-labile subunit of the heterodimeric transcription factor HIF-1, coordinates cellular adaptation to hypoxia through the transcriptional regulation of over 300 genes implicated in angiogenesis, metabolic reprogramming, and cell survival (35). Under normoxic conditions, prolyl hydroxylases (PHDs) employ oxygen and α-KG as co-substrates to hydroxylate HIF-1α, thereby targeting it for ubiquitin-mediated proteasomal degradation via the von Hippel-Lindau (pVHL) E3 ligase complex (36). Direct experimental evidence demonstrates that Fumarate directly suppresses PHD activity through covalent adduction at conserved cysteine residues, a process termed succination, which diminishes the enzymes’ affinity for α-KG. Consequently, HIF-1α accumulates and translocates to the nucleus, where it dimerizes with HIF-1β to initiate the expression of hypoxia-responsive genes (37). In immune cells, succinate accumulation in LPS-activated macrophages was shown to prolong HIF-1α stabilization and enhance IL-1β production (38). Fumarate likely has a similar effect, elevated endogenous fumarate in inflammatory macrophages can sustain HIF-1α, promoting glycolytic metabolism and pro-inflammatory gene programs (39). Loss of FH leads to the accumulation of the fumarate in renal cell carcinomas, which in turn drives HIF-1α stabilization and signaling. Fumarate also reacts with the major cellular antioxidant, GSH, to form a novel metabolite, succinated glutathione (GSF). GSF aberrantly consumes nicotinamide adenine dinucleotide phosphate (NADPH), amplifying mitochondrial reactive oxygen species (ROS) levels, which are necessary and sufficient for HIF-1α stabilization in FH-deficient cells (40). The FH-deficient metabolic shift to aerobic glycolysis leads to heightened ROS production. This increase in ROS provides a second, parallel mechanism for ROS-dependent stabilization of HIF1α, further maintaining a pseudo-hypoxic gene expression profile (41). Besides, when IL-10 is present, it reduces succinate and increases fumarate via boosting SDH, which paradoxically correlates with reduced HIF-1α and IL-1β (6, 7).

#### 4.1.4 NLRP3 inflammasome and pyroptosis

Emerging evidence indicates that fumarate modulates NLRP3 inflammasome activity. Indirect evidence from DMF treatment studies has demonstrated that DMF and related derivatives suppress NLRP3 activation, thereby reducing IL-1β maturation in macrophages (42). Both exogenous DMF and endogenous fumarate accumulation have been reported to induce succination of gasdermin D (GSDMD), although the concentrations and subcellular contexts differ substantially. DMF acts globally at millimolar concentrations, whereas endogenous fumarate promotes GSDMD succination primarily under conditions of FH deficiency or intense metabolic flux. GSDMD functions as the principal effector of pyroptosis, and its succination results in functional inactivation. Importantly, this modification occurs prior to caspase-1/11-mediated cleavage of GSDMD. Experimental evidence indicates that DMF treatment disrupts the interaction between GSDMD and caspase-1 without affecting caspase-1 activation itself. Consequently, succination inhibits caspase-mediated GSDMD activation and subsequent pore formation within the plasma membrane, thereby preventing IL-1β release and pyroptotic cell death (43). In a rat model of bilateral cavernous nerve injury, DMF was shown to attenuate oxidative stress and suppress NLRP3 inflammasome-mediated pyroptosis through activation of the Nrf2/HO-1 pathway, ultimately ameliorating erectile dysfunction (44). Consistent with these findings, *in vivo* administration of a fumarate-enhancing agent reduced IL-1β levels and conferred protection against sepsis, supporting the notion that fumarate serves as an endogenous regulator of hyperactive inflammasome signaling (45). Potential mechanisms may include direct succination of NLRP3 or associated adaptor proteins, as well as modulation of the intracellular redox environment, thereby limiting inflammasome priming. Current evidence predominantly implicates the NLRP3 inflammasome, whereas potential effects of fumarate on absent in melanoma 2 (AIM2) or NLR family CARD domain-containing 4 (NLRC4) inflammasomes remain largely unexplored. This anti-pyroptotic activity is consistent with the broader anti-inflammatory properties attributed to fumarate, particularly under conditions of chronic inflammation.

#### 4.1.5 MAPK/ERK pathways

In renal cell carcinomas characterized by FH deficiency, a subset of tumors exhibits upregulation of the mitogen-activated protein kinase (MAPK)/extracellular signal-regulated kinase (ERK) pathway along with activation of WNT/Notch signaling (46). In MS patient, DMF can interfere with MAPK signaling cascades. Studies indicate that DMF attenuates phosphorylation of both ERK1/2 and p38 MAPK in activated immune cells (47). Furthermore, DMF suppresses p38 MAPK activity, leading to downregulation of vascular endothelial growth factor (VEGF) expression, thereby counteracting high glucose-induced damage in retinal pigment epithelial (RPE) cells (48). A particularly notable action of DMF is its specific inhibition of the ERK1/2 branch within the MAPK cascade. This suppression results in diminished activation of mitogen- and stress-activated kinase 1 (MSK1), a downstream effector. Disruption of the ERK1/2-MSK1 axis represents a key mechanism through which DMF interferes with NF-κB signaling, ultimately promoting an immature dendritic cell phenotype with reduced capacity to activate pro-inflammatory T cells (49). Given the central role of MAPKs in regulating inflammatory mediators such as TNF and interleukin-8 (IL-8), fumarate-mediated dampening of these pathways further reinforces an immunosuppressive milieu.

#### 4.1.6 JAK-STAT pathway

The Janus kinase-signal transducer and activator of transcription (JAK-STAT) pathway constitutes a central signaling axis that transduces signals from cytokines, interferons, and growth factors to the nucleus, thereby regulating inflammation, immune cell differentiation, and cell survival. Dysregulation of this pathway has been implicated in numerous autoimmune and inflammatory diseases, including multiple sclerosis, rheumatoid arthritis, and systemic lupus erythematosus. Emerging evidence indicates that fumarate modulates JAK-STAT signaling through both direct and indirect mechanisms (1). Direct inhibition of JAK kinases by fumarate. In activated B-cell-like diffuse large B-cell lymphoma (ABC-DLBCL) cells, which depend on constitutive STAT3 signaling, DMF treatment potently suppresses STAT3 phosphorylation. Mechanistically, DMF directly succinates Janus kinase 1 (JAK1) at conserved cysteine residues C257 within the FERM domain and C731, as demonstrated by mass spectrometry. Succination at C257 weakens the interaction between JAK1 and the IL-10 receptor, thereby impairing downstream STAT3 activation (50). This direct modification of JAK kinases by fumarate represents a novel mechanism for inhibition of JAK-STAT signaling (2). Indirect modulation via ER stress and upstream signaling. In contrast-induced acute kidney injury, iohexol triggers endoplasmic reticulum (ER) stress, which subsequently activates the JAK2-STAT3 pathway. DMF treatment reduces expression of ER stress markers, including PERK, phosphorylated eIF2α, ATF4, and CHOP, and consequently decreases phosphorylated JAK2 and STAT3 levels, thereby suppressing NLRP3 inflammasome-mediated pyroptosis (51). Moreover, STAT3 knockdown synergistically enhances the inhibitory effects of DMF on pyroptosis, suggesting that DMF acts upstream of JAK-STAT signaling by alleviating ER stress. In addition, fumarate may indirectly suppress JAK-STAT signaling by reducing production of upstream cytokines such as IL-6 and IL-10, as observed in macrophages and T cells. (3) Context-dependent effects and distinction from endogenous fumarate. Most current evidence regarding JAK-STAT modulation derives from pharmacological DMF treatment at supraphysiological concentrations. Whether endogenously accumulated fumarate, such as that observed in FH-deficient cells or LPS-activated macrophages, similarly modifies JAK kinases or alters STAT phosphorylation remains unclear. Direct succination of JAK or STAT proteins by endogenous fumarate has not yet been demonstrated. Future investigations employing unbiased chemoproteomic approaches will be necessary to identify potential direct fumarate targets within the JAK-STAT signaling cascade under physiologically relevant disease conditions.

In summary, Collectively, fumarate exerts pleiotropic immunomodulatory effects by targeting multiple signaling pathways, including NF-κB, NRF2, HIF-1α, JAK-STAT signaling, and inflammasome activation, thereby generating broad anti-inflammatory effects (**Figure 2**). Through coordinated regulation of these interconnected signaling nodes, fumarate promotes immune cell phenotypes that favor inflammatory resolution and immune tolerance rather than persistent pro-inflammatory activation. This mechanistic versatility underlies the therapeutic potential of fumarate across a wide spectrum of immune-mediated disorders, as discussed in subsequent sections.

Beyond its effects on individual pathways, fumarate orchestrates a highly integrated signaling network. For example, NRF2 activation not only coordinates antioxidant responses but also physically interacts with the NF-κB subunit p65 to suppress its transcriptional activity. Similarly, HIF-1α stabilization may potentiate STAT3-dependent gene expression under inflammatory conditions. Future studies should therefore investigate how fumarate-induced post-translational modifications simultaneously reprogram these interconnected signaling cascades to determine the overall inflammatory outcome.

### 4.2 Immune cell-specific roles of fumarate

#### 4.2.1 Macrophages

In macrophages, unbiased metabolomic profiling has identified fumarate as a markedly upregulated metabolite following lipopolysaccharide (LPS) stimulation, accompanied by downregulation of FH and enhanced activity of the argininosuccinate shunt, leading to pronounced cytosolic fumarate accumulation. During early LPS exposure, mitochondrial bioenergetics and FH expression remain largely unchanged, suggesting that induction of argininosuccinate synthase (ASS1) serves as the primary driver of fumarate buildup. Supporting this, both pharmacological inhibition of the pathway using the glutamic-oxaloacetic transaminase 2 (GOT2) inhibitor aminooxyacetic acid and genetic silencing of argininosuccinate lyase (ASL) effectively abolish LPS-induced fumarate accumulation. Although FH suppression at later time points (24–48 h post-LPS) may further exacerbate fumarate accumulation, the distinct regulatory mechanisms governing mitochondrial versus cytosolic FH isoforms, and their relative contributions to fumarate dynamics, require further elucidation (52).

TCA cycle rewiring in activated macrophages is influenced not only by inflammatory stimuli but also by extracellular nutrient availability, particularly glutamine. Mechanistic insights primarily derive from studies on β-glucan–induced trained immunity, which shares key metabolic features with LPS-activated macrophages, including enhanced glycolysis and TCA cycle remodeling. Arts et al. demonstrated that β-glucan training of human monocytes results in marked fumarate accumulation driven by glutaminolysis-the conversion of glutamine to glutamate and subsequently to α-KG, replenishing TCA cycle intermediates via anaplerosis. Pharmacological inhibition of glutaminolysis using BPTES (a specific glutaminase 1 inhibitor) significantly reduced fumarate levels in trained monocytes and abrogated trained immunity, as assessed by cytokine production and H3K4me3 enrichment at pro-inflammatory gene promoters (53). Exogenous fumarate itself recapitulated key epigenetic and functional features of β-glucan training. These findings underscore glutamine flux as a critical determinant of fumarate accumulation in myeloid cells undergoing metabolic reprogramming.

Nevertheless, it must be acknowledged that direct experimental evidence demonstrating that extracellular glutamine deprivation perse alters fumarate accumulation in LPS-stimulated inflammatory macrophages is currently absent from the literature. The studies described above were performed in the context of β-glucan-induced trained immunity, which involves a 24-h primary stimulation followed by a 5-day resting period, rather than acute LPS activation. Whether the same dependency on glutaminolysis for fumarate production operates in the acute inflammatory response to LPS remains to be established. Future studies employing stable isotope-assisted metabolomics under defined nutrient conditions (e.g., glutamine deprivation or supplementation) in LPS-activated macrophages are warranted to quantitatively assess the impact of glutamine availability on fumarate-driven inflammatory outcomes.

**Figure 3:**
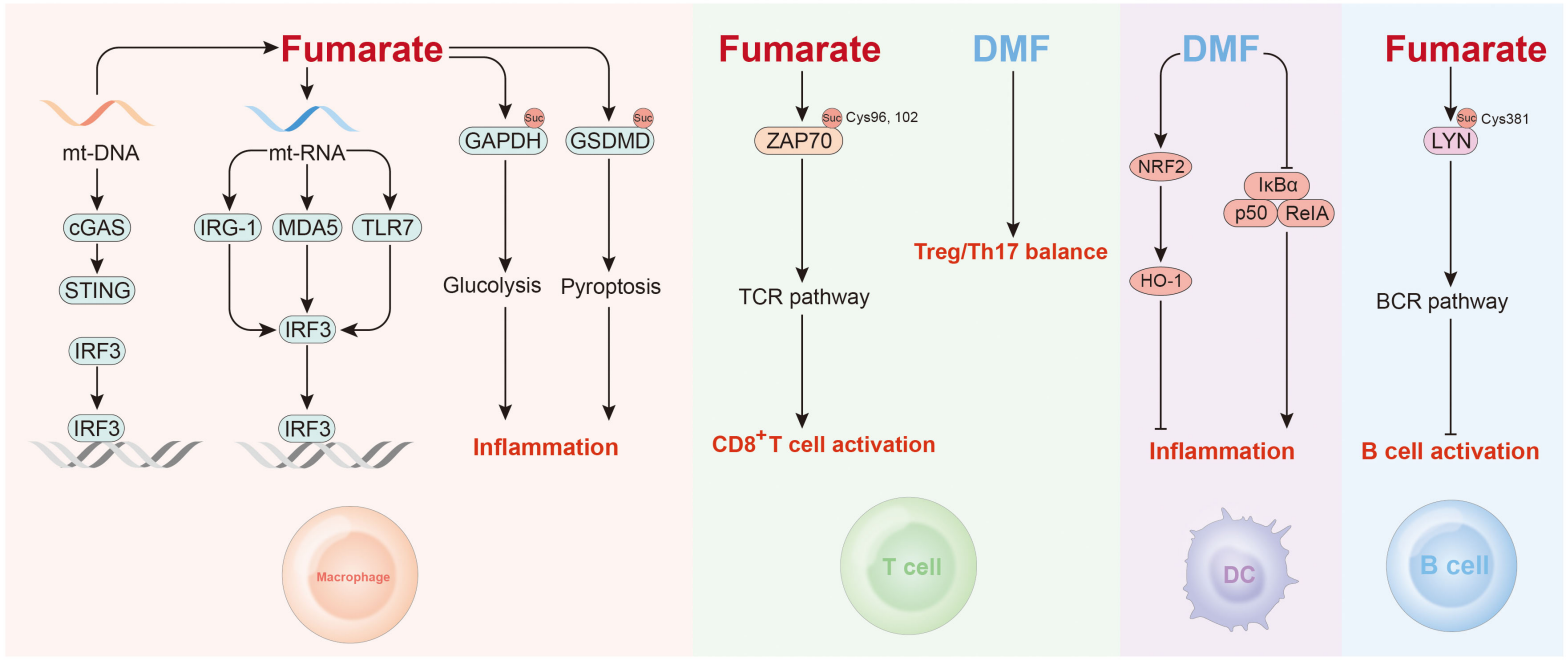
Fumarate and DMF orchestrate immune cell activation and inflammation via targeted cysteine succination across different immune cell types. Fumarate-mediated regulation of immune cell signaling via cysteine succination. Accumulated fumarate modifies key cysteine residues on proteins in different immune cells, including macrophages, T cells, dendritic cells, and B cells, thereby modulating inflammation and adaptive immune responses. In macrophages, fumarate succinates GAPDH and GSDMD, leading to enhanced glycolysis and pyroptosis, while mt-DNA and mt-RNA trigger cGAS/STING and IRF3-mediated inflammatory gene expression. In T cells, fumarate succinates ZAP70 at Cys96/102 to promote CD8_+_ T cell activation. In dendritic cells, dimethyl fumarate (DMF) modulates NRF2/HO-1 and NF-κB signaling to balance Treg/Th17 differentiation and control inflammation. In B cells, fumarate succination of LYN at Cys381 enhances B cell receptor (BCR) signaling, promoting activation. This schematic highlights the cell-type-specific immunomodulatory effects of fumarate and its pharmacological mimic, DMF, through protein succination.

Macrophage-derived fumarate stabilizes HIF-1α (54, 55), promoting glycolytic metabolism and enhancing IL-1β expression. Concurrently, fumarate covalently modifies central metabolic enzymes; for example, succination of glyceraldehyde-3-phosphate dehydrogenase (GAPDH) inhibits glycolysis and redirects carbon flux (52), Impaired FH signaling also activates double-stranded RNA sensors, including retinoic acid-inducible gene I (RIG-I) and melanoma differentiation-associated protein 5 (MDA5), as well as single-stranded RNA sensing via Toll-like receptor 7 (TLR7). Notably, these observations derive from chronic fumarate accumulation due to FH inhibition, distinct from transient fumarate elevations in LPS-activated macrophages. FH-inhibition–induced fumarate accumulation promotes mitochondrial DNA (mtDNA) leakage and activates the cGAS–STING pathway, amplifying inflammation (56) (**Figure 3**) (56).

#### 4.2.2 Dendritic Cells

DCs are another innate immune population modulated by fumarate. DMF restricts pro-inflammatory DC maturation, decreasing expression of costimulatory markers and secretion of IL-12 and TNF, indicative of a tolerogenic phenotype (23, 47). DMF attenuates DC-driven T cell activation by interfering with NF-κB and MAPK signaling. For instance, DMF blocks TLR-induced NF-κB activation by modifying critical cysteine residues on enzymes required for K63 and M1 polyubiquitin chain formation (49), reducing IL-6, IL-1β, and IL-12 production. Simultaneously, DMF enhances anti-inflammatory mediator production, including IL-10, via the NRF2/HO-1 pathway (9) (**Figure 3**). These mechanisms underscore fumarate’s role as a metabolic checkpoint in innate immune activation.

#### 4.2.3 T Cells

Fumarate and DMF profoundly influence adaptive immunity, particularly T lymphocytes. DMF promotes apoptosis and reduces proliferation in activated T cells, sometimes leading to lymphopenia (57). These effects are subset-specific: DMF preferentially diminishes pro-inflammatory Th1 and Th17 cells while preserving or expanding regulatory populations, including Th2 and Tregs. In MS and psoriasis, DMF reduces memory T cells and IFN-γ–producing Th1 and IL-17-producing Th17 populations, while increasing naïve T cells and anti-inflammatory lineages (57, 58). One longitudinal investigation documented substantial declines in interferon-gamma (IFNγ)-producing Th1 and IL-17-producing Th17 CD4_+_ T cells following DMF exposure, whereas the frequency of IL-4-secreting Th2 cells rose (58, 59). Parallel changes occur in psoriasis, where DMF elevates the abundance of forkhead box P3 (FoxP3)_+_ Tregs and reduces IL-17_+_CCR6_+_ Th17 cells (59). This recalibration of the Treg/Th17 balance is therapeutically advantageous in autoimmunity, as Tregs suppress inflammatory processes counteracted by Th17-driven responses. The selectivity arises from differential metabolic vulnerability, DMF induces electrophilic stress to which effector T cells, especially memory subsets, are highly susceptible, whereas Tregs, endowed with elevated antioxidant gene expression, demonstrate greater resilience (60). Consequently, DMF selectively depletes pathogenic T cells while maintaining regulatory integrity, thereby promoting an overall anti-inflammatory shift. Further mechanisms include suppression of inflammatory cytokine secretion by T cells and inhibition of autoreactive T cell infiltration into tissues (58, 61). Together, these actions underscore how fumarate mitigates T cell-mediated chronic inflammation through dual strategies: direct cytolytic effects on hyperactive effector T cells and concurrent strengthening of immunoregulatory pathways. What’s more, In the context of FH-deficient tumors-a distinct setting from pharmacological DMF treatment-fumarate accumulation can reach chronically high levels within the tumor microenvironment. In tumor cells, depletion of FH leads to the accumulation of fumarate within the tumor interstitial fluid. Elevated fumarate can directly mediate succination of zeta-chain-associated protein kinase 70 (ZAP70) at residues C96 and C102, impairing its kinase functionality in infiltrating CD8_+_ T cells. This modification attenuates CD8_+_ T cell activation and dampens antitumor immune responses through T cell receptor (TCR) signal transduction, as demonstrated both *in vitro* and *in vivo (*62) (**Figure 3**).

The metabolic control of CD4^+^ T cell differentiation is an emerging theme, but direct evidence linking endogenous fumarate accumulation to epigenetic remodeling in these cells is limited. Below we synthesize available findings while clearly distinguishing pharmacological from physiological contexts.

Direct biochemical evidence from cell-free and macrophage systems. Fumarate is a structural analog of α-KG and competitively inhibits α-KG-dependent dioxygenases, including JmjC-domain-containing histone demethylases (KDMs) and ten-eleven translocation (TET) DNA demethylases, as demonstrated in cell-free assays and in FH-deficient cells (63, 64). In β-glucan-trained human monocytes, fumarate accumulation via glutaminolysis reduces KDM5 activity and increases H3K4me3 at promoters of pro-inflammatory cytokine genes (e.g., TNFA, IL6) *(*53). This establishes a robust biochemical mechanism, but it has not been directly validated in CD4^+^ T cells.

Evidence from pharmacological fumarate esters (such as DMF/MMF) in T cells. In MS patients treated with DMF, a consistent shift from pro-inflammatory Th1/Th17 toward anti-inflammatory Th2 and Tregs is observed (64, 65). Mechanistically, DMF treatment induces DNA hypermethylation of the *miR-21* promoter in CD4^+^ T cells, leading to reduced miR-21 expression and decreased CC chemokine receptor 6 (CCR6), a chemokine receptor critical for Th17 brain homing (66). Additionally, α-KG – the physiological cofactor for KDMs and TET enzymes – has been shown to promote DNA demethylation and influence Th17/Treg balance in T cells (67). Since fumarate antagonizes α-KG, it is plausible that fumarate could reciprocally inhibit demethylation. However, these findings are derived from supraphysiological drug concentrations (DMF) or from α-KG supplementation, not from endogenous fumarate accumulation. DMF also depletes GSH and directly succinates numerous proteins, effects that may not be recapitulated by endogenous fumarate (58). Absence of direct evidence for endogenous fumarate in CD4^+^ T cells. To date, no study has demonstrated that endogenous fumarate accumulation (e.g., due to FH downregulation or metabolic reprogramming during T cell activation) alters histone methylation, DNA methylation, or the Th17/Treg balance in primary CD4^+^ T cells. Therefore, whether endogenous fumarate regulates CD4^+^ T cell subset differentiation via KDM or TET inhibition remains speculative. Future investigations using T cell-specific FH knockout models or stable isotope-assisted metabolomics under defined nutrient conditions are required to address this critical gap.

In summary, the biochemical capacity of fumarate to inhibit α-KG-dependent demethylases provides a plausible mechanism, but direct evidence in CD4^+^ T cells is lacking. Most clinical immunomodulatory effects observed with DMF likely reflect its pharmacological actions rather than physiological roles of endogenous fumarate. Addressing this critical gap will require T-cell-specific FH knockout models combined with unbiased chromatin profiling (e.g., ATAC-seq, ChIP-seq for H3K4me3/H3K27me3) and stable isotope-resolved metabolomics to directly link endogenous fumarate flux to epigenetic remodeling in CD4^+^ T cell differentiation.

#### 4.2.4 B cells

Both pharmacologically (via DMF) and under conditions of FH deficiency, fumarate can directly succinate and inhibit the Src-family kinase LYN at Cys381, a proximal and indispensable node in B-cell receptor (BCR) signaling. Accumulation of fumarate (via FH inhibition) or treatment with DMF (at pharmacological concentrations) blunts B-cell activation, proliferation, and antibody production *in vitro* and *in vivo*, in part by disabling LYN and thereby attenuating downstream BCR signaling cascades (68) (**Figure 3**). This places fumarate as a covalent, pathway-proximal brake on B-cell activation. Multiple clinical and translational studies in MS patients document consistent, selective remodeling of circulating B-cell subsets with DMF. Within months of therapy, there is a preferential reduction of memory B cells, with relative enrichment of naïve and transitional populations. Importantly, immunoglobulin levels tend to remain stable, suggesting preserved basal humoral immunity despite the memory-cell contraction. These shifts correlate with a less pro-inflammatory B-cell phenotype and may contribute to reduced disease activity (69). DMF has likewise been associated with increased frequencies of regulatory B-cell phenotypes, including IL-10 producing subsets, and with reduced costimulatory capacity, changes that collectively bias toward immune hyporesponsiveness. Such effects have been observed regardless of whether DMF is deployed as first- or second-line therapy, though prior treatments and disease stage modulate the magnitude of these changes (70). At the cellular level, ex vivo exposure of B cells to DMF can induce apoptosis and diminish activation marker expression, reinforcing the notion that DMF acts directly on B cells in addition to system-level immunomodulation (71).

### 4.3 Fumarate immunoregulation via non-enzymatic and enzymatic reactions

A distinctive mechanism through which fumarate modulates immune cell activity is via post-translational modification of proteins, particularly through succinylation or succination. Through a non-enzymatic mechanism, fumarate forms covalent adducts with cysteine thiol groups on target proteins, a modification termed succination, which generates S-(2-succinyl)cysteine (2SC) residues (53, 72, 73). This alteration can significantly affect protein function and is prominently observed in settings of fumarate accumulation. For instance, in FH-deficient cells, succination of Keap1, an inhibitor of the antioxidant transcription factor NRF2, results in Keap1 inactivation and subsequent stabilization of NRF2 (74). Beyond Keap1, succination targets a broad range of proteins; as previously noted with GAPDH, modifications of inflammasome constituents or transcription factors may further influence inflammatory signaling. Additionally, lysine succinylation-an enzymatically catalyzed process utilizing succinyl-CoA, represents another modality of epigenetic regulation. Elevated intracellular levels of fumarate and succinate can inhibit α-KG-dependent dioxygenases, including Jumonji C domain-containing histone demethylases (JMJDs) and TET DNA demethylases (75, 76). Consequently, impaired demethylation leads to hypermethylation of both histones and DNA, constituting an epigenetic shift that can remodel transcriptional programs. While such fumarate-driven epigenetic rewiring is well established in cancer cells bearing FH mutations-resulting in a hypermethylator phenotype, immune cells may also experience analogous changes following transient fumarate elevation, potentially affecting the methylation status of cytokine or immunoregulatory gene promoters (63, 77). Although this field remains under investigation, one study demonstrated that exposure of macrophages to cell-permeable fumarate induced methylation changes at IL-1β and IL-6 gene loci, correlating with transcriptional repression, likely via inhibition of KDMs that normally facilitate their expression (78).

## 5 Fumarate in chronic inflammatory diseases

Research over the past decade has increasingly connected fumarate dysregulation to the pathogenesis of chronic inflammatory and autoimmune diseases. Both mechanistic studies in models and observations in patients implicate fumarate or its metabolism in conditions such as autoimmunity, inflammatory bowel disease, and atherosclerosis.

### 5.1 Autoimmune diseases

Autoimmune pathologies such as MS, psoriasis, and systemic lupus erythematosus (SLE) have provided a valuable context for investigating the immunomodulatory role of fumarate, spurred notably by the clinical application of fumarate-based therapeutics. In T cell–driven conditions like MS and psoriasis, the efficacy of DMF underscores fumarate’s broad influence. Beyond exogenous intervention, emerging research explores how dysregulated endogenous fumarate metabolism may intrinsically contribute to autoimmune pathogenesis. A notable observation comes from SLE, a systemic autoimmune disorder marked by type I interferon overexpression and pervasive inflammation. Monocytes and macrophages derived from SLE patients exhibit diminished expression of FH, resulting in accrual of intracellular fumarate (9, 52). This fumarate buildup correlates with heightened spontaneous release of mitochondrial nucleic acids and consequent activation of interferon signaling pathways. Thus, acquired FH deficiency in lupus may mechanistically underpin the disease’s hallmark interferon-rich inflammatory milieu, a causal link currently under active investigation. In psoriasis, although overt fumarate accumulation has not been consistently detected within lesions, metabolic dysregulation and oxidative stress are evident, and the therapeutic benefit of fumarates implies involvement of pathways modulated by fumarate, including NF-κB activation and Th17 differentiation (79–81). Furthermore, in experimental models such as collagen-induced arthritis and experimental autoimmune encephalomyelitis (EAE), administration of fumarate esters ameliorated disease severity, supporting the notion that fumarate-driven anti-inflammatory mechanisms, such as HO-1 induction and IL-10 upregulation, counteract core autoimmune processes (82). However, it is crucial to recognize that these protective effects are achieved at pharmacological concentrations and may not reflect the physiological or pathophysiological roles of endogenous fumarate. This endogenous fumarate accumulation due to acquired FH deficiency in SLE is pathophysiologically distinct from the pharmacological effects of DMF, although both may converge on interferon pathways.

### 5.2 Inflammatory bowel disease

Inflammatory bowel disease (IBD), which includes Crohn’s disease and ulcerative colitis, is characterized by chronic inflammation of the gastrointestinal mucosa. Emerging research is increasingly exploring the involvement of fumarate in IBD, both from a metabolic standpoint and as a potential therapeutic agent. Metabolomic studies conducted on samples from IBD patients have occasionally identified alterations in TCA cycle intermediates; however, succinate, often derived from microbial and macrophage sources, has received greater attention than fumarate. Preclinical models, nevertheless, offer deeper insight. In murine colitis models, DMF administration exerted protective effects: one investigation observed that DMF mitigated the severity of colitis induced by agents such as dinitrobenzene sulfonic acid (DNBS), with treated animals exhibiting reduced mucosal injury and decreased levels of pro-inflammatory cytokines (83). These beneficial outcomes were linked to DMF’s capacity to suppress NF-κB and NLRP3 inflammasome activation in colonic macrophages, alongside its role in activating NRF2, a pathway known to enhance intestinal barrier integrity. Such findings have spurred proposals to repurpose DMF for IBD treatment (84, 85). Another area of interest is fumarate’s influence on the gut microbiome. Given that intestinal microbes both produce and consume fumarate, oral DMF administration may modify microbial composition, potentially ameliorating IBD through correction of dysbiosis—a hypothesis currently under active investigation (86, 87). In summary, although fumarate has not yet become a central theme in IBD pathogenesis research, preliminary evidence implies that augmenting fumarate-mediated anti-inflammatory signaling, for instance via DMF, could offer a promising strategy for alleviating intestinal inflammation. **I**n contrast to the well-characterized pharmacological effects of DMF, the pathophysiological role of endogenous fumarate accumulation in the inflamed gut mucosa remains largely unexplored.

### 5.3 Atherosclerosis and chronic cardiovascular inflammation

Atherosclerosis is characterized by chronic inflammatory processes within arterial walls, involving key cellular players such as macrophages, foam cells, and T lymphocytes. The potential role of fumarate in this context is increasingly examined from a translational research perspective. Observational data from patients receiving DMF for MS or psoriasis indicate reduced atherosclerotic activity; Some research noted that such treatment correlated with decreased levels of circulating inflammatory monocytes and a trend toward attenuated plaque progression over time (88). Experimental studies in rabbit models of atherosclerosis revealed that DMF administration resulted in smaller lesion size and reduced expression of vascular inflammation markers, an effect attributed primarily to inhibition of NF-κB signaling in macrophages and diminished endothelial oxidative stress (89). Similarly, in diabetic apolipoprotein E (ApoE)-deficient mice, a model exhibiting accelerated atherosclerosis, DMF significantly lowered aortic plaque burden and suppressed expression of TNFα and IL-1β within lesions (90). Mechanistically, fumarate-mediated activation of NRF2 in both endothelial cells and macrophages may promote plaque stability through reduced foam cell formation and downregulation of adhesion molecules that facilitate immune cell recruitment (33, 91, 92). Additionally, by modulating DC function, DMF can attenuate T cell-driven vascular inflammation, while its ability to induce Tregs may further support an anti-atherogenic immune milieu. Although these findings remain preliminary, they collectively highlight fumarate as a promising candidate for therapeutic intervention in atherosclerosis. While dedicated clinical trials targeting cardiovascular end points have yet to be conducted, the concept of modulating vascular inflammation through fumarate-driven immunometabolic pathways continues to gain scientific interest. It is important to note that these atheroprotective effects are derived from DMF treatment and may not be equivalent to the consequences of endogenous fumarate modulation.

### 5.4 Fumarate levels as disease biomarkers

Emerging research further correlates dysregulated fumarate levels with clinical severity and inflammatory activity across various diseases. In SLE, as noted earlier, decreased FH expression in monocytes, and consequent fumarate accumulation, is associated with elevated serum interferon levels and greater disease activity (52). In MS, metabolomic profiling of patient blood has identified perturbations in TCA cycle metabolites during relapse phases, though fumarate itself is rapidly metabolized and seldom accumulates detectably in circulation. Consequently, there is growing interest in employing protein succination, quantified via 2SC adducts, as an indirect biomarker of elevated fumarate exposure. Increased succination of proteins has been documented both in hereditary FH-deficient tumors and within inflammatory skin lesions of psoriasis patients (73, 74). Should robust assays become available, such biomarkers may offer a window into pathological fumarate accumulation or broader metabolic dysregulation in inflammatory conditions. At present, the most definitive evidence stems from scenarios involving genetic or functional FH impairment. For instance, hereditary FH deficiency (as in HLRCC syndrome) not only predisposes to renal cancer but also promotes an inflammatory kidney phenotype characterized by macrophage activation, echoing the inflammatory mechanisms observed in SLE where partial FH loss in immune cells may contribute to pathogenesis. These associations position fumarate as a measurable mediator and potential indicator of inflammatory disease mechanisms and progression.

In summary, current studies across a spectrum of chronic inflammatory disorders recognize fumarate as a pivotal immunometabolic node capable of either exacerbating or alleviating pathology in a context-dependent manner. This context-dependency is influenced by factors such as the source of fumarate (endogenous accumulation vs. exogenous administration), the specific cell type involved, the disease stage, and the duration of exposure (**Table 1**). In autoimmune settings, intrinsic fumarate accumulation, resulting from metabolic alterations, can act as a driver of pathological inflammation, whereas therapeutic potentiation of fumarate signaling generally exerts anti-inflammatory effects. Although the exploration of fumarate modulation remains more nascent in conditions like IBD and atherosclerosis, emerging preclinical evidence supports its therapeutic relevance. Deviations in fumarate homeostasis, whether excessive accumulation, as in FH dysfunction, or insufficient signaling in certain regulatory contexts, are increasingly implicated in the immunometabolic imbalance underlying chronic inflammation. These insights not only rationalize the application of fumarate-based therapeutics but also stimulate interest in novel interventions targeting fumarate-metabolizing pathways.

**Table 1:** Context-dependent roles of fumarate in inflammation.

Cell type/Disease context	Fumarate source	Concentration/duration	Primary target(s)	Signaling outcome(s)	Net effect	Evidence location (section)
LPS-activated macrophages	Endogenous (ASS1-ASL shunt, FH downregulation)	Acute (hours), moderate	PHDs, GAPDH	HIF-1α↑, glycolysis↑, IL-1β↑	Pro-inflammatory	4.2.1, 4.1.3
IL-10-treated macrophages	Endogenous (via SDH↑)	Acute	SDH → fumarate↑	HIF-1α↓, IL-1β↓	Anti-inflammatory (paradoxical)	4.1.3
FH-deficient macrophages (siRNA/pharmacologic inhibition)	Endogenous (FH loss)	Subacute (hours–days), high	mtDNA, cGAS-STING	Type I IFN↑, IRF3↑, inflammation↑	Pro-inflammatory	4.2.1
β-Glucan-trained monocytes	Endogenous (glutaminolysis)	Chronic (days), moderate	KDM5 (histone demethylase)	H3K4me3↑ at TNFA/IL6 promoters, TNF/IL-6↑	Pro-inflammatory (trained immunity)	4.2.1
FH-deficient RCC (HLRCC) – tumor cells	Genetic (constitutive FH loss)	Chronic (years), very high	KEAP1, PHDs, KDMs, PTEN	NRF2↑, HIF-1α↑, hypermethylation, AKT↑	Mixed (pro-tumorigenic/pseudo-hypoxic)	4.1.3, 4.1.5, 4.3
Tumor-infiltrating CD8+ T cells (HLRCC)	Endogenous (cancer-cell-derived fumarate)	Chronic (weeks–months), high (TIF)	ZAP70 (C96/C102)	TCR signaling↓, activation↓, cytotoxicity↓	Anti-inflammatory/immunosuppressive	4.2.3
B cells (FH inhibition or DMF treatment)	Endogenous (FH loss) or exogenous (DMF)	Subacute, moderate–high	LYN (C381)	BCR signaling↓, proliferation↓, antibody production↓	Anti-inflammatory/immunosuppressive	4.2.4
SLE patient monocytes/macrophages	Endogenous (acquired FH↓)	Chronic (months–years), moderate	Mitochondrial nucleic acid release	cGAS-STING↑, type I IFN↑	Pro-inflammatory	5.1
MS patient T cells (DMF therapy)	Exogenous (DMF/MMF)	Chronic (weeks–months), supraphysiologic	KEAP1, IKKβ, GSDMD, ERK1/2	NRF2↑, NF-κB↓, GSDMD inactive, Th1/Th17↓, Th2/Treg↑	Anti-inflammatory	4.1.1, 4.1.2, 4.1.4, 4.1.5, 4.2.3
Psoriasis patient T cells (DMF therapy)	Exogenous (DMF)	Chronic (weeks–months), supraphysiologic	Th17/Treg axis	Th17↓, Treg↑, IL-17↓	Anti-inflammatory	4.2.3
DCs (DMF treatment)	Exogenous (DMF)	Acute–subacute, supraphysiologic	CBM complex (ubiquitination), ERK1/2, MSK1	NF-κB↓, maturation↓, IL-12/TNF↓, tolerogenic phenotype	Anti-inflammatory/tolerogenic	4.2.2
EAE/collagen-induced arthritis models (DMF)	Exogenous (DMF)	Subacute–chronic, supraphysiologic	HO-1, IL-10, NF-κB	HO-1↑, IL-10↑, disease severity↓	Anti-inflammatory	5.1
DNBS-induced colitis (DMF treatment)	Exogenous (DMF)	Subacute (days), supraphysiologic	KEAP1, NLRP3	NRF2↑, NLRP3↓, IL-1β↓, mucosal protection↑	Anti-inflammatory	5.2
Atherosclerosis models (DMF treatment)	Exogenous (DMF)	Subacute–chronic, supraphysiologic	NF-κB, NRF2, adhesion molecules	NF-κB↓, NRF2↑, foam cell↓, plaque burden↓	Anti-inflammatory	5.3
HTLV-1-infected T cells (DMF)	Exogenous (DMF)	Acute, supraphysiologic	CARD11-BCL10-MALT1 (CBM)	NF-κB↓, proliferation↓	Anti-inflammatory	4.1.1
Renal cell carcinoma (FH deficiency)	Genetic (FH loss)	Chronic (years), high	KEAP1, PHDs	NRF2↑, HIF-1α↑, aerobic glycolysis↑	Mixed (pro-tumor)	4.1.3, 4.1.5
Contrast-induced acute kidney injury (DMF)	Exogenous (DMF)	Acute, supraphysiologic	ER stress (PERK/eIF2α), JAK2-STAT3	ER stress↓, JAK2-STAT3↓, NLRP3 pyroptosis↓	Anti-inflammatory	4.1.6
ABC DLBCL cells (DMF)	Exogenous (DMF)	Acute, supraphysiologic	JAK1 (C257, C731)	STAT3 phosphorylation↓	Anti-inflammatory	4.1.6
Bilateral cavernous nerve injury (DMF)	Exogenous (DMF)	Subacute, supraphysiologic	NRF2/HO-1, NLRP3	NRF2↑, NLRP3↓, pyroptosis↓	Anti-inflammatory	4.1.4

Each row represents a distinct setting from the main text (Sections 4–5). Fumarate source is categorized as endogenous (metabolic rewiring), endogenous/FH loss (genetic or acquired FH deficiency), or exogenous (DMF/MMF, supraphysiologic). Concentration/duration indicates relative levels and time scales (acute, subacute, chronic). Primary target(s) are validated or strongly implicated molecular targets. Net effect is classified as pro-inflammatory, anti-inflammatory, immunosuppressive, or mixed. All entries are directly derived from cited studies; no speculative claims are included. See indicated sections for original references and detailed mechanisms.

## 6 Clinical research progress and applications of fumarate-related drugs

The immunomodulatory properties of fumarate have been translated into clinical therapies, most notably DMF. Originally developed for psoriasis and later approved for MS, DMF and related fumarate derivatives are now being explored in other inflammatory conditions. This section highlights the clinical advancements and mechanistic insights for fumarate-based drugs, we also summarize the information on fumarate derivatives that have been approved for clinical treatment and are currently undergoing clinical trials. (**Table 2**) It should be noted that all mechanisms described in this section refer to pharmacological concentrations of DMF/MMF and do not necessarily reflect endogenous fumarate physiology.

**Table 2:** The use of fumarate derivatives in clinical treatment.

Drug name	Brand name	Clinical use or trial	Regulatory status	Level of evidence	Ref
Dimethyl fumarate (DMF)	Tecfidera®	Approved for Relapsing forms of multiple sclerosis (MS)	FDA 2013, EMA 2017	Phase III RCT	(93, 94)
Dimethyl fumarate (DMF)	Skilarence®	Approved for Moderate-to-severe plaque psoriasis	EMA 2017	Phase III RCT	(95)
Fumaric acid esters mixture (FAE)	Fumaderm®	Approved for Severe plaque psoriasis	Germany 1994	Phase III RCT	(96)
Diroximel fumarate (DRF)	Vumerity®	Approved for Relapsing forms of MS	FDA 2019	Phase III RCT	(97)
Monomethyl fumarate (MMF)	Bafiertam®	Approved for Relapsing forms of MS	FDA 2020	Phase III RCT	(98)
Dimethyl fumarate (DMF)	Unknown	Clinical Trial for Amyotrophic lateral sclerosis (ALS)	Australia & Europe (multi-centre) 2018 (Phase II)	Phase II completed	(99)
Dimethyl fumarate (DMF)	Unknown	Clinical Trial for COVID-19 in hospitalised adults	UK (RECOVERY Trial) 2021	Platform trial	(100)
Fumaric acid esters (Fumaderm®)	Fumaderm®	Clinical Trial for Cutaneous lupus erythematosus	Germany 2014 (Phase II)	Phase II study	(33)

Clinical development and regulatory approval status of fumarate-based therapeutics. The table summarizes currently approved fumarate-derived drugs and fumaric acid ester formulations, including their brand names, therapeutic indications, regulatory approval status, and levels of clinical evidence. In addition, ongoing or completed clinical investigations have explored the therapeutic potential of fumarate compounds in amyotrophic lateral sclerosis (ALS), COVID-19, and cutaneous lupus erythematosus, highlighting the expanding translational relevance of fumarate-targeted therapies across inflammatory, autoimmune, and neurodegenerative diseases.

### 6.1 DMF in multiple sclerosis

DMF (brand name *Tecfidera^®^*) is one of the major success stories of repurposing a metabolic compound for immune therapy. It was FDA-approved in 2013 as an oral treatment for relapsing-remitting MS, after Phase III trials showed significant efficacy (94). In two pivotal studies, oral DMF reduced the annualized relapse rate of MS by 44–53% compared to placebo and lowered the risk of disability progression. MRI outcomes were also improved, with far fewer new or enlarging lesions in DMF-treated patients (94). These impressive results established DMF as a first-line MS therapy. Clinically, DMF helps control the aberrant immune attacks on myelin that characterize MS. Mechanistically, as discussed, DMF skews the peripheral immune cell repertoire: it reduces pro-inflammatory memory T cells and B cells while sparing naive and regulatory cells (57). Concurrently, DMF suppresses the production of inflammatory cytokines. including IL-17 and IFN-γ, by T cells and may foster a neuroprotective milieu via NRF2-dependent antioxidant mechanisms within central nervous system (CNS) cells. Within microglia and CNS-infiltrating macrophages, DMF is thought to promote an anti-inflammatory, M2-like polarization, partially mediated through NRF2 and HO-1 induction. Regarding safety, the most frequently reported adverse effects include flushing and gastrointestinal disturbances; its immunomodulatory actions may also lead to lymphopenia (101). In rare instances, prolonged and severe lymphopenia has been associated with opportunistic infections such as progressive multifocal leukoencephalopathy (PML), necessitating periodic monitoring of lymphocyte counts (102). Despite these risks, DMF maintains a favorable risk-benefit profile in MS management. Current research endeavors include comparative studies between DMF and newer fumarate-based formulations, efforts to identify biomarkers predictive of treatment response, and exploration of its potential utility in progressive MS subtypes, motivated by its hypothesized neuroprotective properties, though efficacy in these forms remains unestablished. Collectively, the use of DMF in MS illustrates how targeted modulation of immunometabolic pathways can achieve meaningful control of autoimmune neuroinflammation.

### 6.2 DMF in psoriasis

Long before its use in MS, fumarate therapy was pioneered in psoriasis – a chronic inflammatory skin disease. In fact, a fumarate ester mixture (Fumaderm^®^) containing DMF has been used in Germany since the 1990s for moderate-to-severe psoriasis (81, 88). The favorable outcomes in psoriasis helped spark interest in DMF for other diseases. In 2017, an oral DMF-only formulation (Skilarence*^®^*) was approved in Europe for psoriasis (81). Clinically, DMF induces remission or significant improvement in many psoriatic patients, reducing the psoriasis area and severity index (PASI) score. Its mechanism in psoriasis appears similar to MS-an immune shift from Th1/Th17 dominance toward a more regulated immune profile (59). In skin, DMF reduces the inflammatory infiltrate and the expression of cytokines like IL-17, IL-22, and TNF, which drive keratinocyte activation (103). Notably, DMF increases circulating and lesional Tregs in psoriasis patients, contributing to disease control (59). It may also directly impact skin-resident cells: studies show DMF can inhibit keratinocyte production of chemokines and downregulate endothelial adhesion molecules, thereby reducing immune cell recruitment to the skin. Clinical research has progressed to optimizing fumarate therapy for psoriasis, for example, using slow dose escalation to improve tolerability of gastrointestinal (GI) side effects, or combining DMF with topical therapies. There is also interest in biomarkers, including neutrophil-to-lymphocyte ratio or specific cytokine levels, to gauge DMF response in psoriasis. While biologic drugs, such as TNF or IL-17 inhibitors, are now common in psoriasis, DMF remains an important oral option, especially in patients who prefer an oral systemic agent or have comorbid MS. Ongoing trials are evaluating DMF in other dermatoses and as a maintenance therapy due to its immunomodulatory, rather than outright immunosuppressive, effect.

### 6.3 Inflammatory bowel disease

Small trials or case series are examining DMF in ulcerative colitis and Crohn’s disease. Given the positive results in colitis animal models, researchers speculate that DMF’s combined antioxidant and anti-inflammatory actions might induce remission in IBD (83, 85). An open-label study in ulcerative colitis patients, with mild-to-moderate disease, showed trends of improved endoscopic scores on DMF, although robust controlled data are lacking. A challenge is that DMF’s GI side effects overlap with IBD symptoms, complicating its use. Nonetheless, proof-of-concept trials are underway to see if a certain subset of IBD patients, perhaps those with concurrent psoriasis or axial arthritis, conditions DMF treats could benefit.

### 6.4 Rheumatologic diseases

Fumarates have been evaluated in preclinical models of rheumatoid arthritis (RA), demonstrating therapeutic potential (104). In a murine model of RA, DMF ameliorated joint inflammation and curtailed bone erosion, with observations of reduced IL-1β and IL-6 levels in articular tissues (105, 106). Considerable interest exists in exploring DMF application among RA patients presenting with psoriatic features, as well as its use adjunctively with methotrexate (107). Likewise, SLE, characterized by interferon-associated inflammation, represents a candidate disease for fumarate-based intervention, particularly in light of recent findings indicating FH downregulation in lupus macrophages.

## 7 Future research directions and prospects

Elucidating the causal mechanisms underlying immunometabolic interactions requires rigorous investigation to determine whether abnormalities in fumarate metabolism directly drive chronic inflammation. Key questions remain: does targeted reduction of FH in specific immune cell populations suffice to induce autoimmune pathology? Conversely, can restoration of FH activity, thereby preventing fumarate accumulation, ameliorate disease manifestations? Genetic models will be instrumental in addressing these questions. For instance, mice with cell type–specific FH deletion could clarify how cell-autonomous fumarate dysregulation contributes to systemic autoimmunity. Findings from such models will reflect chronic, high-level fumarate accumulation, which may differ from the effects of acute metabolic rewiring or pharmacological DMF treatment. In human studies, isotope tracing using labeled fumarate or its precursors may help determine whether immune cells actively accumulate fumarate during disease states and whether this metabolic shift precedes clinical exacerbations. Establishing causal inference is essential for validating therapeutic targeting of fumarate-related pathways.

Fumarate operates within a complex metabolic network that includes succinate, malate, itaconate, and other immunomodulatory metabolites. Future investigations should elucidate how fumarate-mediated signaling intersects with these related pathways. For example, both succinate and fumarate accumulate in inflammatory macrophages, yet it remains unclear whether their effects on inflammatory pathways are redundant, additive, or antagonistic. Itaconate, which exhibits anti-inflammatory properties and shares protein-succinating activity with fumarate, raises additional questions: could simultaneous elevation of both metabolites produce synergistic anti-inflammatory effects, or might they compete for molecular targets such as KEAP1? Moreover, fumarate’s link to amino acid metabolism via the aspartate-argininosuccinate shunt implies cross-talk with nitrogen handling and arginine metabolism, warranting exploration of how this interplay influences immune polarization. This is particularly relevant as arginine metabolism supports both nitric oxide and polyamine synthesis in macrophages. Another promising avenue involves examining the interaction between fumarate and energy-sensing systems, including AMPK and mTOR. Fumarate accumulation during TCA cycle impairment may trigger energy stress responses, potentially activating AMPK or NRF2. Systems biology approaches, such as integrated metabolomic and transcriptomic analyses, will be essential for mapping system-wide changes following experimental manipulation of fumarate levels in immune cells. Ultimately, a holistic understanding of immunometabolic networks will identify critical regulatory nodes for therapeutic intervention, potentially informing combination strategies—for instance, concurrently enhancing fumarate signaling while inhibiting succinate activity—to achieve finely tuned immunomodulation.
